# Development an Immune-Related MicroRNA Risk Index in Hepatocellular Carcinoma

**DOI:** 10.1155/2022/5224434

**Published:** 2022-04-12

**Authors:** Shun Zhou, Jian Xu, Dong Wang, Yong Wang, Lijuan Meng

**Affiliations:** ^1^Hepatobiliary Center, The First Affiliated Hospital of Nanjing Medical University, Nanjing, China; ^2^Department of Laboratory Medicine, The First Affiliated Hospital of Nanjing Medical University, Nanjing, China; ^3^Department of Geriatric Oncology, The First Affiliated Hospital of Nanjing Medical University, Nanjing, China

## Abstract

**Purpose:**

Hepatocellular carcinoma (HC) has emerged as one of the most prevalent malignancies on a global scale. Recently, immunotherapy has achieved favorable effectiveness in the management of multiple cancers. However, there are limited therapeutic options for advanced HC. As the liver is a special immune organ, we intend to uncover potential and effective immunotherapeutic modalities for HC. Our study was designed to develop specific immune-related miRNAs (IRMs) for outcome assessment and individualized strategies for the management of HC.

**Methods:**

The miRNA-seq and survival data of TCGA-LIHC dataset was enrolled into this program. We first collected IRMs from Immune-miR website. Differentially expression analysis was applied to screen aberrantly expressed IRMs. In order to set up an IRM-related index (IRMRI) in HC, we conducted the Cox relevant methods. Next, the statistical approaches (survival curve and ROC curve analyses) were utilized to detect the evaluation capacity of our IRMRI. Subsequently, we obtained the target genes of hub miRNAs from IRMRI through three miRNA-related predictive online tools (miRDB, miRTarBase, and TargetScan websites).

**Results:**

Five IRMs were determined to develop the IRMRI. It can effectively segregate all HC cases from two different risk subgroups. We identified a marked discrepancy in survival outcome between the two groups by survival analysis and confirmed the reliability of IRMRI in two testing sets. Moreover, we collected 10 hub target genes (ESR1, IGF1, PDGFRB, JUN, MYC, ZWINT, MAD2L1, TOP2A, KIF11, and CDCA8) which were strongly linked to HC progression and malignant behavior.

**Conclusion:**

We screened out five hub IRMs with clinical value and constructed a risk index model in HC, which can precisely assess the risk status and outcome of patients to a certain extent.

## 1. Introduction

Hepatocellular carcinoma (HC) is the sixth most common malignancy in the world, with more than 850,000 new cases each year [1]. HC ranks second among cancer-related causes of death and its incidence is increasing year by year. As a highly aggressive malignancy, HC kills 750,000 patients worldwide each year [2]. The incidence of HC has a large geographical heterogeneity, with approximately 85% of HC occurring in developing countries and regions and 72% of HC occurring in Asia [3]. Although there are many studies on the development of HC, it is still the tip of the iceberg for understanding its mechanisms. Currently, there are several well-defined risk factors for HC, including cirrhosis, virus infection, high alcohol intake, and aflatoxin B1. It is hard to diagnose HC given its insidious onset in early clinical stage [4]. Serum AFP test and imaging are the most common clinical tests, but these methods have limitations in the diagnosis of early stage. The survival outcome of HC is dismal owing to the tendency of metastasis and the unsatisfactory curative effect [5].

The immune microenvironment (IME) is a medium for the formation of immunocytes infiltrating in tumor tissues. Generated by tumor cells in their struggle with the immune system, IME is the condition and basis for tumor immune escape [6]. HC has unique self-protection mechanisms to evade host immune surveillance, such as secretion of immunosuppressive cytokines, abnormal expression of antigens, and alteration of the local IME [7]. For instance, TGF-*β* has a dual role in tumorigenesis. It blocks tumor cell viability and induces cell apoptosis in the early stage of disease, while it exerts an immunosuppressive role in the late stage of cancer. In HC, abnormal elevation of TGF-*β*1 suppresses the innate immune response and disrupts the antitumor immune response, which in turn facilitates tumor progression [8].

Immunotherapy can alter the function or number of immune cells, the expression of immune receptors or ligands and cytokine levels to achieve antitumor immunity. Immunotherapy strategies currently used for liver cancer include vaccines, immune checkpoint inhibitors, and passaged cell transplantation, which have been shown to be safe and effective [9].

MicroRNAs (miRNAs) are a class of small endogenous RNAs of approximately 18-25 nucleotides in length without protein-coding capacity. By binding with mRNAs, miRNAs could block protein translation at the posttranscriptional level [10]. Although a blood fetoprotein test is more widely used to diagnose HC, it is not very accurate. Elevated fetoprotein often indicates progressive disease. It is urgent to exploit new markers for early diagnosis of HC [11]. Indeed, miRNAs released from human blood by tumor are stable. In addition, circulating miRNAs are highly tolerant to RNA enzyme activity [12]. Recently, Zhou et al. indicate that a collection of seven miRNAs can distinguish HC from healthy and cirrhotic groups with the promise of being an indicator for early diagnosis of HC [13]. miRNAs can be utilized not only for the diagnosis of HC but also for determining prognosis survival. A cohort study including a large sample of patients revealed that HC patients with lower expression of miR-26 presented a dismal survival outcome, suggesting that miR-26 can be applied to assess the outcomes of HC cases [14]. Nevertheless, the clinical potency of immune-related miRNA (IRM) in HC needs to be thoroughly analyzed.

In our academic research, we unearthed the prognostic value of IRMs and set up a risk index of HC based on IRMs which could effectively forecast risk status and survival outcome of HC samples. In the future, personalized treatment for the HC will benefit from our constructed risk index.

## 2. Methods

### 2.1. Data Preparation

In this project, the HC-associated dataset with RNA transcriptome data were collected from TCGA website. Based on the processing of the miRNA data, 375 HC cases were enrolled into our research. We also acquired 374 HC samples with complete mRNA data. In addition, the clinical traits and survival data were obtained from the UCSC Xena source. Each sample in the HC cohort with follow-up time < 30 days were removed. And a total of 245 IRMs were extracted from the Immune-miR database.

### 2.2. Determination of Differentially Expressed miRNAs (DEMs) and mRNA (DEGs)

Difference analysis was performed to collect DEMs and DEGs between HC and normal specimens by “limma” package in R [15]. We selected DEIRMs and DEGs on the basis of |logFC| > 1 and adj-*p* < 0.05 as a filter.

### 2.3. Development of the Prognostic Index

To set up an IRM-related index (IRMRI), HC cases were equally and randomly divided into two sub-cohorts (training cohort and validation cohort). We first employed univariate analysis to screen possible IRMs showing dramatically prognostic ability in the training set. Then, the IRMs were processed by multivariate analysis to generate corresponding coefficients for IRMRI.

The risk value of IRMRI = ∑exp(IRMs)∗coef. The coef is the coefficient of each model IRMs.

### 2.4. Functional Enrichment Analysis

The target genes of miRNA were collected by three predictive online tools (miRDB, miRTarBase, and TargetScan websites). Next, the intersection target genes were collected by overlapping with the list of DEGs. R package “clusterProfiler” was instrumental in uncovering potential function of these intersection genes [16].

### 2.5. Identification of Hub Gene

STRING is an online tool for examining protein-protein interactions (PPI) [17]. An interaction network was created by introduction of the target genes into STRING and visualized via Cytoscape tool [18]. Subsequently, we used CytoHubba algorithm to determine hub genes from the network based on degree score. At the same time, the prognostic power of target genes was detected by the Kaplan-Meier (KM) method.

### 2.6. Statistical Analysis

All statistical data was processed by R v.3.8.2. KM survival method contributed to assess the prognosis difference between two groups. The specificity and reliability of the index model were checked using ROC curves. Moreover, the Cox regressions were utilized to examine the independent value of clinical outcome.

## 3. Results

### 3.1. Determination of DEIRMs and DEGs

**Figure 1:**
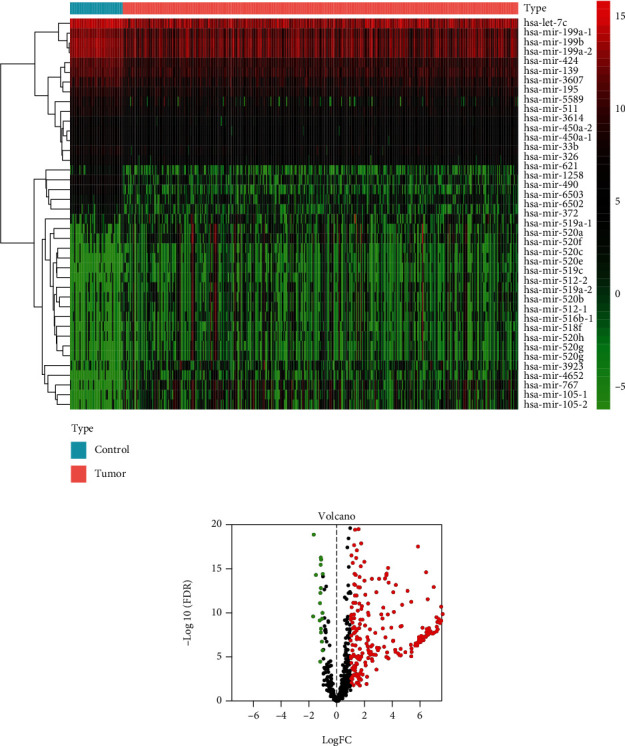
Determination of differentially expressed immune-related miRNAs (DEIRM). (a) Volcano plot of DEIRM in LIHC. (b) Heatmap of DEIRM.

A total of 251 DEMs (229 upregulated and 22 downregulated) between HC and normal specimens were unearthed (Figures 1(a) and 1(b)). According to the 251 DEMs, we further identified 72 DEIRMs by overlapping with IRM set. Additionally, a total of 6219 DEGs (1349 downregulated and 4870 upregulated) were obtained on the basis of 375 HC cases and 50 health controls.

### 3.2. Identification of Prognostic IRMs

After processing prognosis data from the TCGA-HC, 184 HC cases were randomly assigned into the training set. In order to characterize the IRMs with prognostic value, univariate regression was conducted in the training set. We observed that a total of eight IRMs were tightly bound up with clinical outcome (Table 1).

### 3.3. Development of the IRMRI

**Figure 2:**
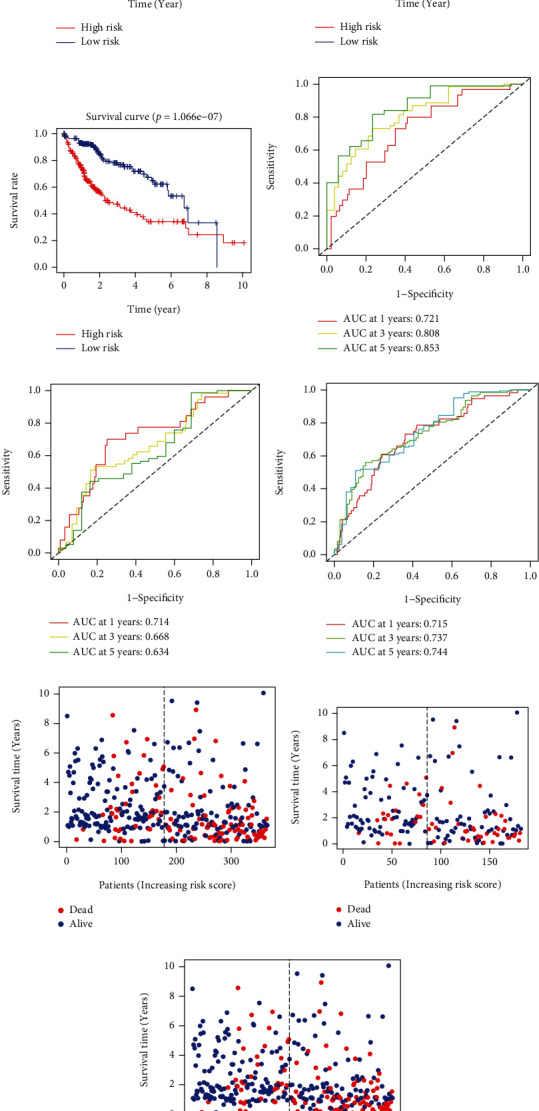
Predictive capability of the IRMS. (a–c) Survival outcome between the two groups in three sets. (d–f) ROC analyses for the IRMS in three sets. (g–i) The clinical outcome of LIHC cases in three sets.

**Figure 3:**
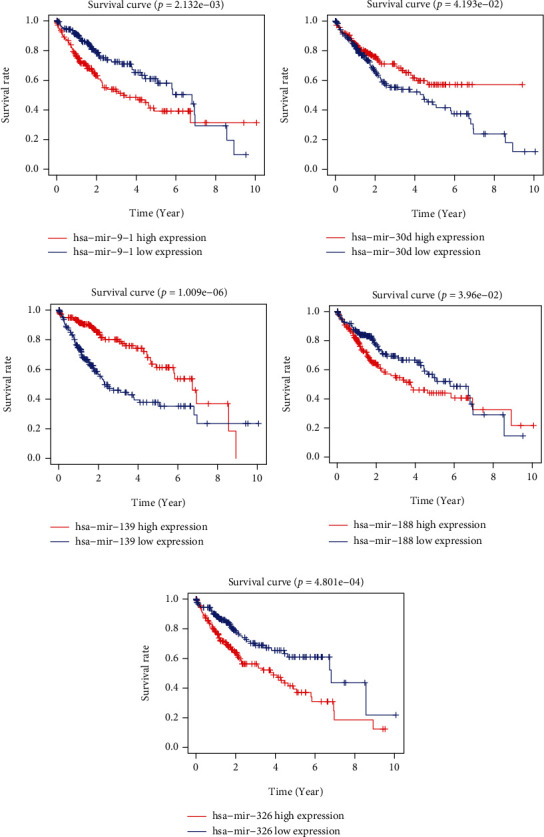
Prognostic value of five hub miRNA. (a) has-miR-9-1. (b) has-miR-30d. (c) has-miR-139. (d) has-miR-188. (e) has-miR-326.

We further carried multivariate analysis to create a prognostic index including five IRMs. The risk score = [hsa − miR − 139 × (−0.36)] + [hsa − miR − 9 − 1 × (0.12)] + [hsa − miR − 30d × (−0.26)] + [hsa − miR − 326 × (0.16)] + [hsa − miR − 188 × (0.33)]. All HC samples were classified into high- and low-risk groups based on the median risk power. As shown in (Figures 2(a) and 2(c)), IRMRI-high group showed worse patient outcome, while IRMRI-low cohort presented favorable clinical outcome. The results of ROC analysis indicated that AUC values were 0.721, 0.808, and 0.853 for 1-, 3-, and 5-year survival, respectively (Figure 2(d)). Meanwhile, we employed same analysis to confirm the capability of the IRMRI by validation cohorts. The results disclosed that there are similar results in the validation set as in the training set (Figures 2(e) and 2(f)). Furthermore, the risk diagram explicitly illustrated the clinical outcome between the two groups (Figures 2(g)–2(i)). We also found that high expression levels of hsa-miR-9-1, hsa-miR-188, and hsa-miR-326 indicated a shorter patient survival time, while low expression levels of hsa-miR-30d and hsa-miR-139 revealed dismal outcome (Figure 3).

### 3.4. Independence Analysis of the IRMRI

**Figure 4:**
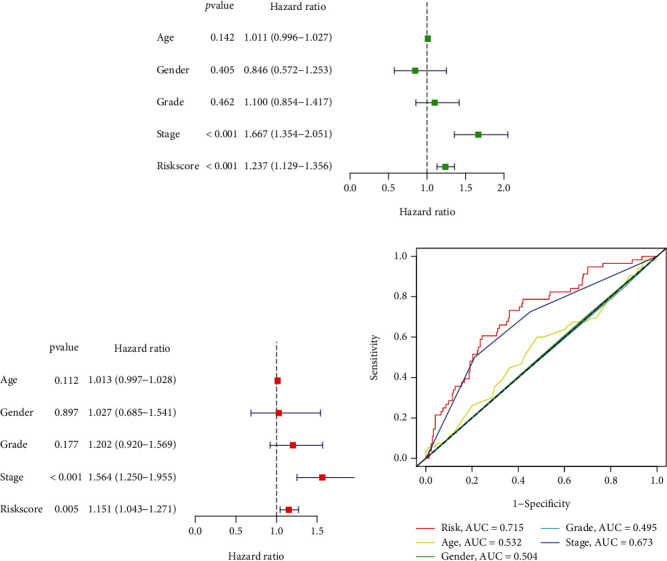
Independent prognosis analysis. (a) Univariate Cox regression. (b) Multivariate analysis for the IRMS. (c) ROC curves of the risk and other traits.

We undertook an intensive analysis of clinical traits on age, gender, stage, and grading of HC cases to estimate the independent performance of the IRMRI. Univariate and multivariate methods both suggested that IRMRI was tightly related to patient outcome (Figures 4(a) and 4(b)). As indicated by Figure 4(c), the AUC of risk score was higher than other clinical factors, indicating a superior predictive power.

### 3.5. Functional Enrichment Analysis

**Figure 5:**
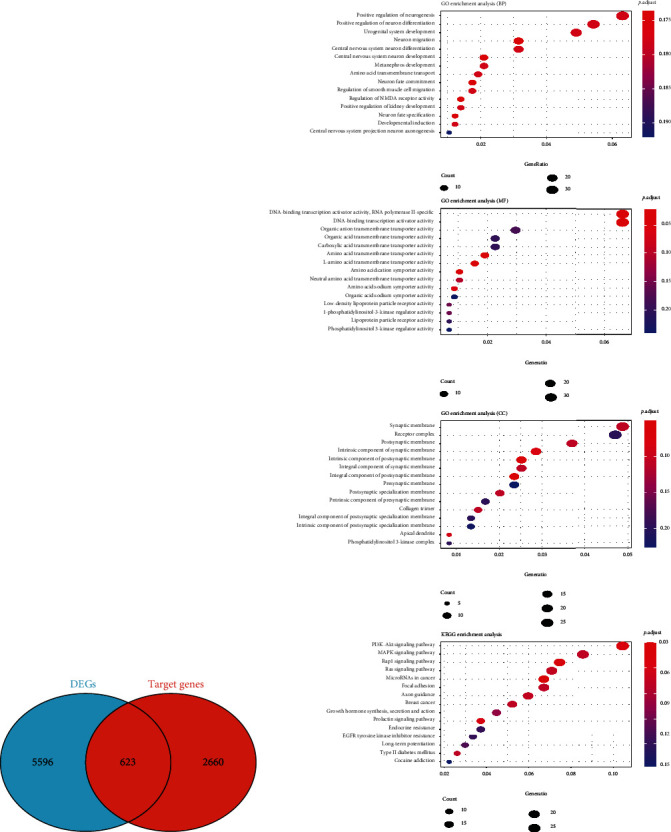
Functional enrichment analysis. (a) Venn diagram of DEGs and target genes. (b) GO analysis (BP). (c) GO analysis (MF). (d) GO analysis (CC). (e) KEGG enrichment analysis.

There were 3,283 target genes determined by three miRNA-related databases. We performed further analysis by making predictions in three databases and taking target genes that were predicted in more than two databases. A total of 623 overlapping genes were then screened to detect the underlying function and pathway (Figure 5(a)). In terms of GO, we observed that neurogenesis regulation, neuron differentiation, and urogenital development were greatly activated. As for KEGG, PI3K/Akt signaling, MAPK signaling, and Rap1 signaling markedly enriched (Figure 5(b)).

### 3.6. Identification of Hub Gene

**Figure 6:**
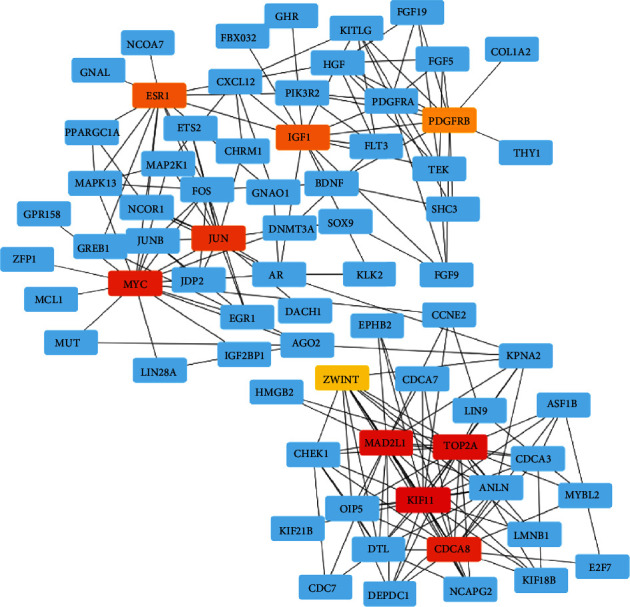
Construction of target gene-based PPI.

**Figure 7:**
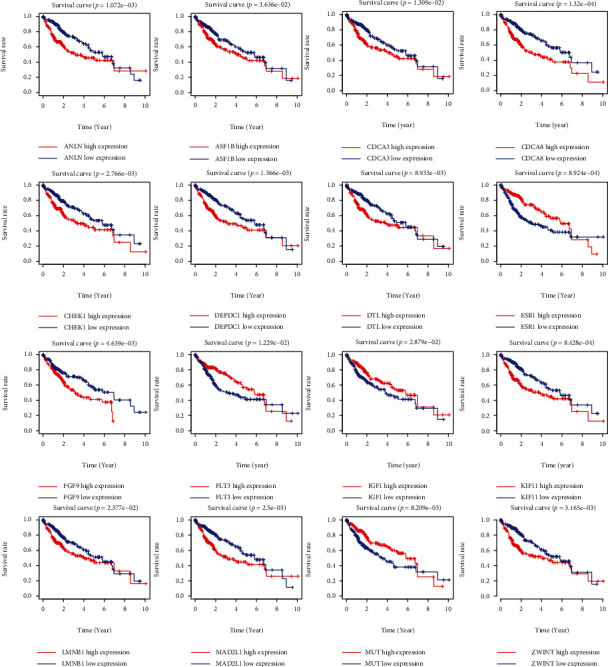
Survival analysis of target genes.

According to the degree score generated by CytoHubba algorithm, we collected top 10 hub genes (ESR1, IGF1, PDGFRB, JUN, MYC, ZWINT, MAD2L1, TOP2A, KIF11, and CDCA8) from the PPI network (Figure 6). Moreover, a total of 16 target genes including FLT3, ANLN, ASF1B, CDCA8, CDCA3, CHEK1, DEPDC1, DTL, ESR1, FGF9, MUT, MAD2L1, KIF11, LMNB1, IGF1, and ZWINT were closely associated with survival outcome of HC sample (Figure 7).

## 4. Discussion

HC is a classic inflammation-related cancer, and the IME plays a central part in the pathogenesis of HC [19]. IME is considered to be a key feature of cancer since the alterations in the IME are involved in all stages of malignant progression from the initial transformation stage to invasion and metastasis. Immunotherapy aims to provide more effective tumor cell targeting by enhancing existing tumor-specific immune responses [20].

In recent years, immunotherapy has been employed as an effective curative strategy for a variety of tumors, including HC [21]. In particular, therapies targeting immune checkpoints have achieved success and improved the clinical outcome of HC cases [22]. However, only a minority of patients benefit from immunotherapy due to the immunosuppressive status in IME [19]. Considering the prominence of IME in cancer progression, investigators should concentrate on uncovering new immune biomarkers and targets for HC management which can offer a reference for early diagnosis and prognosis determination.

Gene immunotherapy has become a promising approach for tumor treatment by restoring the function of tumor suppressor genes or stimulating the production of antitumor immune responses [23]. One of the effective medical methods is immunocytokine therapy which can be achieved by transfecting cytokines such as IL-2 directly in tumor and adjacent tissues [24]. Moreover, the clinical application of immune checkpoint inhibitors opens up new mindsets for HC management. Immune checkpoints that have been extensively analyzed in relation to HC immune escape include PD-1/PD-L1 [25]. PD1 expressed on T cells, B cells, and natural killer (NK) cells could bind with PD-LI and PD-L2 ligands, inhibiting antigen-specific T cell activation and blocking the immune response of T cells in IME [22].

miRNAs have critical biological functions and their altered expression can contribute to cancer progression. Numerous reports have highlighted that miRNAs can regulate tumor initiation and progression as either pro- or anticancer factors [26]. The vast majority of HC can originate from cirrhosis of the liver due to various causes. As various etiologies lead to persistent liver injury and regeneration, individual HC etiologies also result in differential miRNA expression [27]. Hepatitis C virus infection is an integral factor in the pathogenesis of HC. Serum miRNA-27a may be used as an indicator of hepatitis C virus-induced HC [28]. In addition, Cao et al. found that upregulated miRNA-182-5p boosts HC initiation and progression [29]. Nevertheless, the regulatory role of IRMs in malignant behavior of HC needs a more exhaustive investigation.

In this work, an IRM-based prognostic index (has-miR-9-1, has-miR-30d, has-miR-139, has-miR-188, and has-miR-326) was created to analyze the risk status and survival outcome for HC cases. Our established IRMPI has turned out to display optimal independence with respect to survival outcome of patients. Survival analysis can dramatically differentiate the survival prognosis of the two groups. Meanwhile, we applied ROC analysis to detect the predictive reliability of IRMPI. Given the prominence of mRNA-miRNA interactions, we also determined the target genes of five model miRNAs and obtained 10 hub genes (ESR1, IGF1, PDGFRB, JUN, MYC, ZWINT, MAD2L1, TOP2A, KIF11, and CDCA8) with high relevant scores.

Reviewing the previous literature, we found that these model miRNAs are more or less implicated in the formation of various tumors. As suggested by Wong CC et al., miR-139 could serve as a suppressor in HC and upregulation of miR-139 could inhibit the metastatic behavior of HC cell through suppression of ROCK2 [30]. NETA1 could increase the expression of TGF-*β* to facilitate HC cell viability through binding with miR-139 [31]. Also, miR-139 could mediate cell growth and metastasis in HC by targeting Wnt/TCF-4 axis [32]. As a promising noninvasive indicator in breast cancer (BC), miR-188 has been shown to regulate BC cell viability and metastasis by interacting with IL6ST [33]. In glioma, Li N et al. suggested that miR-188 might suppress cell cycle and cell development by inhibiting *β*-catenin [34].

Moreover, we found that among the 10 hub genes, 6 genes (CDCA8, ESR1, MAD2L1, KIF11, IGF1, and ZWINT) had significant prognostic value. Meanwhile, these 6 key genes are tightly bound up with tumor aggravation. For instance, Jeon et al. suggested that CDCA8 could regulate HC cell viability and stemness by targeting Akt/*β*-catenin signaling [35]. Aresti et al. detected the ERS1 expression in lung cancer and normal specimens and indicated its potential ability in evaluating survival outcome [36]. The function of MAD2L1 is related to cell mitosis. As revealed by Li et al., miR-200c-5p could block HC cell growth and survival by binding with MAD2L1 [37]. Similar to our results, Huang et al. also demonstrate that IGF1 could be the carcinogenic factor and possible target in HC by bioinformatic method and experiments [38]. Both KIF11 and ZWINT can affect HC progression by mediating cell proliferation [39, 40].

The signaling pathway is a cellular reaction to signal reception and integration, which modulates gene expression and affects cell viability and apoptosis. Tumor-associated pathways engage in host of cellular activities and metabolic regulation. The activation of these pathways could contribute to the development of tumor malignant behavior and treatment failures [41]. Therefore, therapeutic strategies targeting signaling pathways have been the focus of cancer research, and various targeted inhibitors with promising benefits are becoming prevalent.

PI3K/Akt signaling pathway is mainly modulated by multiple genes such as PTEN, SHIP, and CTMP. PTEN blocks the dephosphorylation of PIP3 to PIP2, which could decrease the expression level of cellular PIP3 in cells and suppress the activation of Akt and its downstream molecules [42]. The downstream regulatory targets of the PI3K/Akt signaling include mTOR, Foxo, and GSK-3. Among them, the mTOR protein complex is a pivotal member. Akt can trigger mTOR kinase activity by negatively regulating the mTORC1 protein complex TSC1-TSC2, which mediates biological processes such as cell cycle, DNA damage repair, and glycogen synthesis [43]. In HC, Jiang et al. reported that PRMT9 could confer powerful migration ability to cancer cells by triggering Akt/GSK-3*β* pathway [44].

MAPK can be stimulated by mitogens, cytokines, and neurotransmitters to mediate cellular signals and exert biological effects by regulating cell growth, apoptosis, and autophagy. The MAPK pathway is composed of a conserved three-tier kinase pattern, including MKK, MKK, and MAPK, which can be triggered sequentially and act on downstream molecules, such as c-Jun and ATF2/6, to control the expression of specific genes and thus adjust the cell viability and differentiation [45].

Up to now, five parallel MAPK signaling pathways have been identified, including ERK1/2, SAPK, ERK5 p38, and JUK. Although each pathway is highly specific, in some cases, there is some crosstalk between them. ERK is mainly a transmitter of cell proliferation signals, while JNK and p38MAPK are mainly activated by various extracellular stimuli, causing a complex series of cellular stress transduction [46]. Several studies have revealed that MAPK signaling remains active at both transcriptional and translational levels in chemotherapy resistance HC cells [47]. Abnormal activation of MAPK pathway is responsible for the loss of cell differentiation and apoptosis, triggering abnormal cell proliferation and malignant transformation, and plays a central part in the mechanism of tumor drug resistance. In addition, upregulated ERK can promote cell division and proliferation by facilitating the cell cycle to enter S phase. Researchers have discovered that Ras mutations in cells induced abnormal upregulation of downstream MEK and ERK, resulting in abnormal proliferation of tumor cells [48].

In summary, we created a five IRM-based prognostic index which could play a central part in survival assessment for HC samples. In the future, our proposed index model might be a valuable informative tool offering clinical guide in HC management.

## Figures and Tables

**Table 1:** General characteristics of prognostic IRM.

miRNA symbol	HR	95% CI	*p* value
hsa-mir-139	0.648	0.538-0.781	5.65E-06
hsa-mir-34a	0.762	0.591-0.981	0.0352
hsa-mir-105-1	1.101	1.029-1.177	0.0049
hsa-mir-9-1	1.145	1.043-1.257	0.0044
hsa-mir-30d	0.740	0.577-0.949	0.0178
hsa-mir-195	0.801	0.658-0.975	0.0275
hsa-mir-326	1.257	1.012-1.562	0.0383
hsa-mir-188	1.336	1.007-1.772	0.0444

## Data Availability

Public data were analyzed in this project. All data can be collected from TCGA database (https://portal.gdc.cancer.gov/).
